# Changes of Causal Attribution by a Co-actor in Situations of Obvious Causality

**DOI:** 10.3389/fpsyg.2020.588089

**Published:** 2021-01-06

**Authors:** Kazuki Hayashida, Yu Miyawaki, Yuki Nishi, Shu Morioka

**Affiliations:** ^1^Department of Neurorehabilitation, Graduate School of Health Sciences, Kio University, Koryo, Japan; ^2^Department of Rehabilitation, Fujiikai Rehabilitation Hospital, Osaka, Japan; ^3^Research Fellow of Japan Society for the Promotion of Science, Tokyo, Japan; ^4^Department of Rehabilitation Medicine, Keio University School of Medicine, Tokyo, Japan; ^5^Neurorehabilitation Research Center, Kio University, Koryo, Japan

**Keywords:** causal attribution, passing responsibility, self-serving bias, co-actor, diffusion of responsibility

## Abstract

In social contexts, people are responsible for their actions and outcomes. Diffusion of responsibility is a well-known social phenomenon: people feel less responsible when performing an action with co-actors than when acting alone. In previous studies, co-actors reduced the participant’s responsibility attribution by making the cause of the outcomes ambiguous. Meanwhile, it is unclear whether the presence of co-actors creates diffusion of responsibility even in situations where it is “obvious” that both oneself and the co-actor are the causes of an outcome. To investigate this potential diffusion of responsibility, we used a temporal binding (TB) task as a measure of causal attribution. Low TB effects indicate the enhancement of external attribution (i.e., diffusion of responsibility) in perceptual processing for the action and outcomes. To investigate the influence of presence of a co-actor on causal attribution, participants were required to act under two experimental conditions: an ALONE condition (participant only) or a TOGETHER condition (with a co-actor). The only difference between the two conditions was whether the actions were shared. In addition, to make participants feel responsible, they were induced to feel guilt. In the High-harm condition, participants gave a financial reduction to a third party. When guilt was induced, participants showed lower TB effects in the TOGETHER condition compared to the ALONE condition. Our study suggests that actions with a co-actor change causal attributions even though the causes of the outcome are obvious. This may have implications for understanding diffusion of responsibility in inhumane situations.

## Introduction

People are responsible for their actions and outcomes in the social contexts of human society, and responsibility varies according to various social situations. People feel less responsible when performing an action as a group than when acting alone. This diffusion of responsibility was first proposed by Darley and Latané (1968). Groups tend to make riskier choices than alone (Bradley, 1995), behave more aggressively (Meier and Hinsz, 2004), and have the effect of reducing stress on difficult decision-making (El Zein et al., 2019). On the other hand, diffusion of responsibility has social and moral importance because it may constitute a form of moral disengagement that has been purported to explain inhumane actions (Bandura, 1999).

The most troubling example is “passing the buck”: blaming someone or making them responsible for a problem that one should deal with oneself. Passing the buck (responsibility) to others often causes problems of a social or legal nature. A previous study of violent crimes found that groups reported feeling less guilt than alone (Deschenes and Esbensen, 1999). Diffusion of responsibility has recently been identified as a problem in medical settings, which could affect one’s survival (Henriksen and Dayton, 2006; Christensen, 2018; Mcintosh, 2019). What these situations have in common is that the presence of a “co-actor” enhances attribution of responsibility to the other person in the situations of actions and the outcomes shared because the cause of the outcomes becomes ambiguous.

This ambiguousness of the cause with a co-actor enhances cognitive biases that change one’s internal (self) or external (other people) attributions. One of the famous examples is the self-serving bias; people tend to attribute causes of negative outcomes more to external factors than to themselves (Miller and Ross, 1975; Mezulis et al., 2004). A previous study reported the influence of co-actors on changing causal attributions in a dice-tossing gambling task (Li et al., 2010). In the alone condition, participants tossed only one die, while in the co-actors condition, the other two dice were tossed by other players. In this procedure, the co-actors reduced participants’ control over the outcome by two-thirds because of the sharing of actions and outcomes. Subjective ratings of attribution results showed that the participants felt more responsible for monetary loss in the alone condition than in the co-actors condition. These results supported the concept of self-serving bias.

In the procedure of the previous study, performing with co-actors made the cause of the outcomes ambiguous. Meanwhile, it is unclear whether the presence of co-actors causes diffusion of responsibility even in situations where it is “obvious” that both oneself and the co-actor are the causes of the outcomes. This potential diffusion of responsibility (or passing responsibility) may explain inhumane behavior in groups. Beyer et al. (2017) experimented on the effect of diffusion of responsibility in situations where the cause of the outcomes can be distinguished from the self or another using a task requiring performance of timing of actions. In this procedure, however, the outcomes of actions were independent because the actions of self and the other were different in time. This procedure may be unable to investigate the mechanism of attribution of responsibility to others in particular situations where actions and outcomes are shared.

In the present study, we investigated whether the presence of a co-actor potentially changes internal or external attributions in situations where the causes of the outcomes were not ambiguous. In situations where it is obvious that both oneself and the co-actor are the cause of the outcomes, subjective measurement of the cause of the outcomes could show cognitive bias. Therefore, we used a recently developed method that can implicitly and quantitatively measure causal attribution: temporal binding (TB). In the TB task, participants are required to estimate the time interval between their actions (pressing the key) and the outcomes (tone). The perceived shorter time interval is used as an index of the greater TB effect. It has recently been agreed that in TB, closer time perception of action and its outcome indicates a greater perceptual causal attribution (Buehner and Humphreys, 2009, 2010; Cravo et al., 2009; Suzuki et al., 2019). Takahata et al. (2012) reported that using a method combining a simple gambling task and a TB task that associated a tone and a reward, a negative financial outcome reduced the TB effect. The results of TB were consistent with self-serving bias theory of causal attribution. We therefore used TB as an implicit measure of causal attribution in this study.

The purpose of this study was to investigate whether the presence of a co-actor potentially changes internal or external attributions by using an implicit measure of causal attribution in situations where the causes of the outcomes were obvious. To test this prediction, we used a modified TB task combined with a simple financial gambling game. To investigate the influence of diffusion of responsibility with the presence of a co-actor, participants were required to act under two experimental conditions: an ALONE condition (participant only) or a TOGETHER condition (with a co-actor). In addition, to make participants feel responsible, they were induced to feel guilt. Participants, therefore, gave a financial reduction to a third party (High-harm condition). Compared with the Baseline condition that involved no financial reduction, a high effect of TB would indicate enhancement of internal attribution and a low effect of TB would indicate enhancement of external attribution. We predicted that, in the High-harm condition, compared to the Baseline condition, internal attribution of the actions and outcomes would be enhanced in the ALONE condition; on the other hand, external attribution would be enhanced in the TOGETHER condition.

If diffusion of responsibility can occur even in obvious situations, we suggest that the coping strategies for suppressing inhumane actions by diffusion of responsibility may be different from what we have previously understood. Previous studies have suggested that ambiguous situations can lead to diffusion of responsibility; thus, to cope with difficulties based on diffusion of responsibility, one must avoid ambiguous situations (that is, obvious situations). However, if diffusion of responsibility can occur even in obvious situations, this coping strategy may not be correct. We believe that the findings of this study will be important for understanding the mechanism of diffusion of responsibility and discussing how to cope with difficulties.

## Materials and Methods

### Participants

Differences in outcome caused by the manipulation of social context in the presence of a co-actor condition has not been investigated in previous TB research. Given that a previous study investigating TB in relation to negative emotions employed sample sizes of 16 or 17 participants (Yoshie and Haggard, 2013), we aimed for a minimum sample size of 18. To allow for dropouts, we tested 24 right-handed healthy volunteers. The experimenter and the participants did not know each other prior to the experiment. The data of one participant were lost because of technical failure. The data of two other participants were excluded from data analysis because their mean TB values were >3SD from the group mean. Two participants spontaneously expressed suspicion about a third party in the task in the post-experimental questionnaire, and their data were excluded from analysis as well. Thus, the data of 19 participants (16 females, mean age = 20.473, SD = 1.263) were included in the analyses. All participants reported normal vision, hearing, and verbal and finger function needed for the experiment. The Kio University ethics committee approved the study’s procedures (R1-29), and the researchers conducted the experiment in accordance with the Declaration of Helsinki.

### Apparatus

The task and measuring system were created using Laboratory Virtual Instrument Engineering Workbench (National Instruments). A 19-inch display (Mitsubishi RDT191VM, Japan) and a keyboard (DELL RT7D60 Microsoft comfort curve keyboard 3000, Japan) were used to conduct the experimental task.

### Measures and Procedure

First, participants completed a preliminary practice TB task to familiarize themselves with the experimental TB task. The goal of the TB task was to measure participants’ perceptions of time intervals between an action and its outcome (tone). The time interval between a key press with the right index finger and the subsequent tone (50 ms) was 1–1000 ms. In ms units, participants verbally estimated the time interval between the key press and the tone. This task was administered to participants over 20 trials. The time intervals were random for each trial, and after estimating the time interval, each participant received feedback on the actual time interval. Because this task’s purpose was participant training, it was excluded from subsequent analyses.

Next in the experimental TB task, we set up a situation in which participants were induced to feel guilty when they feel responsible for harming others (Baumeister et al., 1994). To induce guilt, we used a situation in which participants gave a financial reduction to a third party (Wagner et al., 2012). The participants were provided with an explanation that this was an experiment involving a financial reduction for a third party who was not present in the laboratory. The third party had already participated in the other experiment and received some money. The participants were additionally explained that the amount of the reward that the third party would receive was determined by this experiment. The participants were instructed that they and the third party did not know each other. To investigate the influence of the presence of a co-actor, the participants experienced the two conditions in randomized order. The ALONE condition required the key to be pressed by the participant alone; the TOGETHER condition required the key to be pressed simultaneously with the experimenter. In both, the ALONE and TOGETHER conditions, the experimenter was located next to the participant.

**FIGURE 1:**
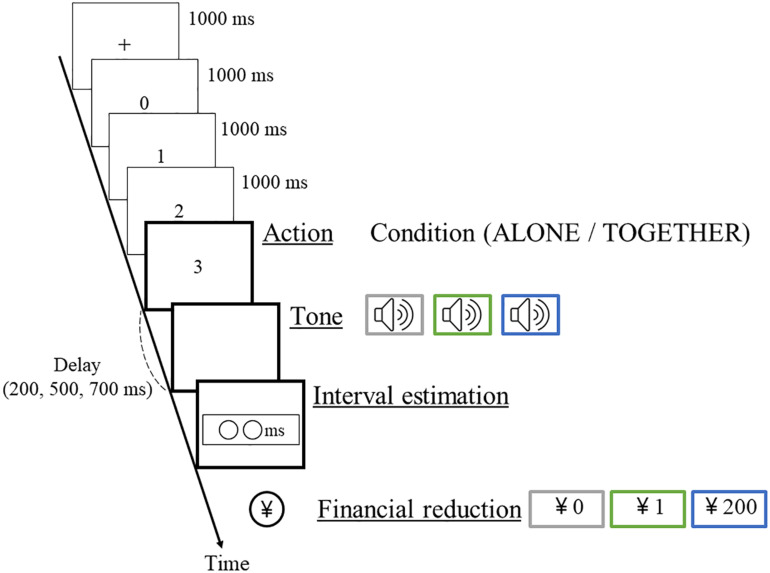
Experimental paradigm. The ALONE condition required the key to be pressed by the participant alone; the TOGETHER condition required the key to be pressed simultaneously with the experimenter. After a black cross was presented on screen for 1 s, the number was counted every 1 s, and when the number displayed 3, participants were instructed to press the key. Participants estimated the time intervals and answered the values using a keyboard. The financial reduction value was associated with each pitch. The financial reduction consisted of three conditions: the Baseline condition, the Low-harm condition, and the High-harm condition. When the pitch of the Low-harm condition or the High-harm condition sounded, participants actually performed the procedure to reduce the amount of money after estimating the time interval.

After a black cross was presented on the screen for 1 s, the number was counted every 1 s, and when the number displayed 3, participants were instructed to press the key. The time intervals (delay) of the experimental task were, randomly, 200, 500, or 700 ms (actual time interval); however, participants knew only that the interval was random, from 1 to 1000 ms. Participants estimated the time intervals and answered the values using a keyboard. The pitch of the tone consisted of 300, 1000, or 3000 Hz, and the financial reduction value was associated with each pitch. The financial reduction consisted of three conditions: the Baseline condition with no reduction, the Low-harm condition with a reduction of 1 yen, and the High-harm condition with a reduction of 200 yen. The Low-harm condition was set to assess whether the amount of money in the High-harm condition was appropriate for inducing guilt. The valences of the outcome (i.e., the pitch) were pre-determined in the task and had nothing to do with participants’ responses. Pairs of pitch and financial loss were counterbalanced across all participants. The order of the pitch of the tone was unpredictable. The giving of unpredictable outcomes to the third party was a gambling task for participants. This reduction value was determined based on a preliminary experiment with reference to a previous study (Takahata et al., 2012). When the pitch of the Low-harm condition or the High-harm condition sounded, participants actually confirmed the procedure to reduce the amount of money by the experimenter after estimating the time interval. The procedure confirmed the participant’s obvious expectations, serving to bolster the veracity of the bogus feedback. Participants were not informed about how much the third party had and how much money would be reduced. Participants performed 81 trials (nine trials per each actual time interval, three actual time intervals, and three conditions) each in the ALONE condition and the TOGETHER condition (Figure 1). After 10 practice trials, participants confirmed that they fully understood the task, including the association between the pitch of the tone and the financial reduction value. Actually, the key used by the experimenter did not react in the TOGETHER condition, and the amount of money was not reduced. Participants were informed of these facts after the completion of all experiments.

A previous study showed a correlation between TB effects and personality questionnaires, reporting that more empathetic individuals showed a more dramatic reduction in TB effects when their actions had more rather than less harmful outcomes (Caspar et al., 2016). Data from a number of standard questionnaires, including the Big Five personality (Namikawa et al., 2012) and the Interpersona1 Reactivity Index (Sakurai, 1988), were available from data collection occurring prior to participation. The order of the questionnaires was counterbalanced across participants.

In two post-session emotion self-reports, participants were asked to rate how guilty they felt during the experiment in the Low-harm and High-harm conditions. Participants were also asked to describe other feelings during the experiment. The first self-report was about how guilty the participants felt throughout the experiment on a scale ranging from 1 (not very guilty) to 7 (very guilty) (self-report 1: S1). The other self-report was about whether one felt more guilty in the ALONE condition or in the TOGETHER condition on a scale ranging from 1 (very guilty in the TOGETHER condition) to 7 (very guilty in the ALONE condition) (self-report 2: S2). We asked participants to rate their relative feelings, to detect any differences between the conditions.

### Statistical Analyses

First, analysis using the Kolmogorov–Smirnov test showed that all the TB data were normally distributed. We analyzed the TB effect using a two-way analysis of variance (ANOVA) with condition (ALONE and TOGETHER) and negative outcome (Baseline, Low-harm, and High-harm) as the within-subject factors. The Bonferroni method was used for multiple comparisons. The TB effect values used for the ANOVA in each negative outcome were the means of 27 trials pooled across the three action-outcome delays (actual time interval).

The third party was the same person across all trials, and the participant’s guilt about the third party could potentially increase as the number of trials increased in the High-harm condition. To test this possibility, a trial to trial time-series analysis needed to be performed. Linear mixed effects models were used to analyze time-series changes in estimated time intervals in the High-harm condition using the trial number, the actual time interval of each trial, and their interaction as fixed effects, and subject as random effects, in both the ALONE and TOGETHER conditions (see Supplementary Data used for the analysis). In this analysis, no significant effects of trial number indicate that the increasing guilt did not cause the TB effect to systematically increase or decrease over time.

A Wilcoxon signed-rank test was used for post-session self-reports. Additionally, the relationship between the TB effect and post-session self-reports in the High-harm condition was analyzed using Spearman’s rank correlation coefficient.

A multiple regression model was used to explore the association of causality and empathy or personality. The time intervals in the High-harm condition were selected as the dependent variables, while each subscale of the Interpersonal Reactivity Index and the Big Five Inventory were used as the independent variables, respectively. *P*-values of less than 0.05 were considered statistically significant. SPSS Statistics for Windows ver. 24 (IBM, Japan) was used as the analysis software. The datasets presented in this study can be found in the Supplementary Material.

## Results

**FIGURE 2:**
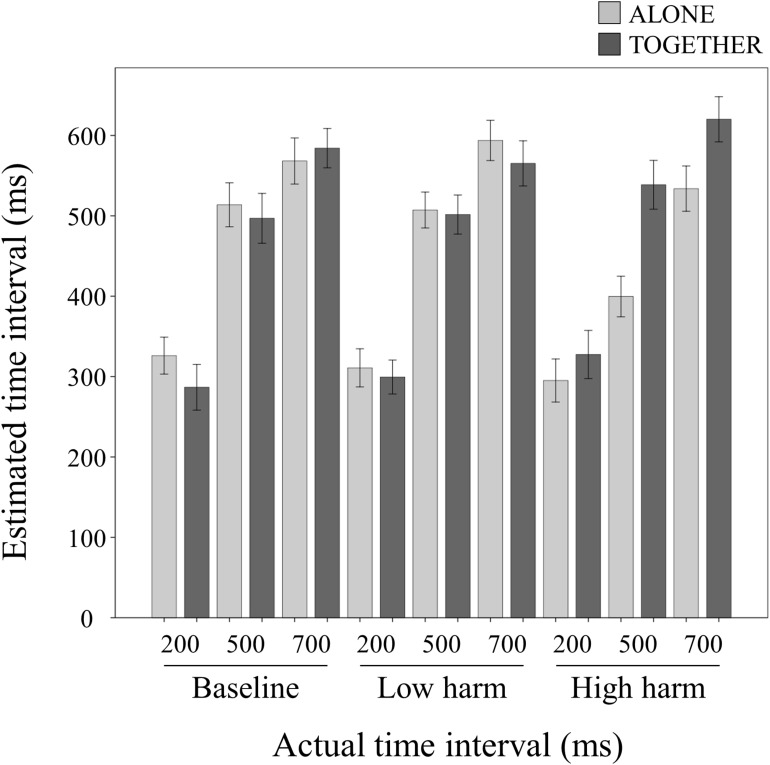
The estimated time interval in each actual time interval. Estimated action-tone intervals (ms) in the ALONE and TOGETHER conditions and negative outcome conditions. Data represent means ± standard error.

**FIGURE 3:**
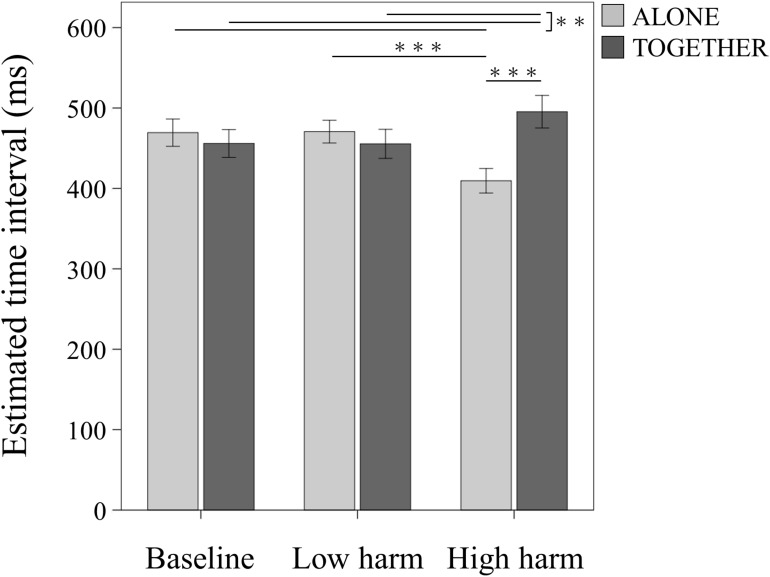
Results of the estimated time interval. Estimated action-tone intervals (ms) in the ALONE and the TOGETHER conditions and negative outcome conditions, pooled across the three action-outcome delays. ^∗∗∗^*p* < 0.001, ^∗∗^*p* < 0.01. Data represent means ± standard error.

Compared to the Baseline conditions, a shorter time interval, that is, an enhanced TB effect, indicates enhancement of internal attribution, and a longer time interval, that is, a reduced TB effect, indicates enhancement of external attribution. Figure 2 shows the estimated time interval in each actual time interval of each condition. The interaction between condition and negative outcome value was significant (*F*(2, 36) = 15.356, *p* < 0.001, *$\eta_{p}^{2}$* = 0.460). In the High-harm condition, a simple effect was a significantly lower time interval in the ALONE condition than the TOGETHER condition (*F*(1, 18) = 18.535, *p* < 0.001, *$\eta_{p}^{2}$* = 0.507), and no significant differences were observed in the Baseline condition (*F*(1, 18) = 1.013, *p* = 0.327, *$\eta_{p}^{2}$* = 0.053) and the Low-harm condition (*F*(1, 18) = 1.024, *p* = 0.325, *$\eta_{p}^{2}$* = 0.054). A simple effect in the ALONE condition was significant (*F*(2, 36) = 11.880, *p* < 0.001, *$\eta_{p}^{2}$* = 0.398). In the ALONE condition, multiple comparisons revealed that the time interval in the High-harm condition was significantly lower than in the Baseline condition (mean difference = −59.817, 95%CI = −94.458 to −25.174, *p* = 0.001) and the Low-harm condition (mean difference = −61.114, 95%CI = −89.061 to −33.166, *p* < 0.001), and no significant differences were observed in the Baseline and Low-harm conditions (mean difference = −1.297, 95%CI = −28.438 to 25.843, *p* = 0.921). A simple effect in the TOGETHER condition was significant (*F*(2, 36) = 10.610, *p* < 0.001, *$\eta_{p}^{2}$* = 0.370). In the TOGETHER condition, multiple comparisons revealed that the time interval in the High-harm condition was significantly higher than in the Baseline condition (mean difference = 39.480, 95%CI = 15.810–63.148, *p* = 0.002) and the Low-harm condition (mean difference = 39.953, 95%CI = 17.049–62.856, *p* = 0.001), and no significant differences were observed in the Baseline and Low-harm conditions (mean difference = 0.473, 95%CI = −14.616 to 15.562, *p* = 0.948) (Figure 3). A *post hoc* power analysis using G^∗^Power 3.1.9.2 was performed based on the current sample size and effect size. We confirmed that the sample size was enough to achieve a power of 95% at 5% alpha level.

In the time-series analysis, in the ALONE condition, there were no significant effects of trial number (*p* = 0.64) and the interaction between trial number and actual time interval of each trial (*p* = 0.18), and there was a significant effect of actual time interval of each trial (*p* < 0.001). In the TOGETHER condition, there were no significant effects of trial number (*p* = 0.74) and the interaction between trial number and actual time interval of each trial (*p* = 0.54), and there was a significant effect of actual time interval of each trial (*p* < 0.001).

In the post-session self-reports, the subjective ratings of guilt in the High-harm condition (median [IQR], 6 [5–7]) was significantly higher than in the Low-harm condition (median [IQR], 3 [1–5]) (*Z* = −3.645, *p* < 0.001). In the ALONE condition against the TOGETHER condition, the mean difference of the subjective ratings in the High-harm condition (median [IQR], 6 [5–7]) was significantly higher than in the Low-harm condition (median [IQR], 5 [4–6]) (*Z* = −3.401, *p* < 0.001) (Table 1).

**TABLE 1:** Subjective ratings of guilt.

Emotion self-reports	Condition		*p*-values
	
	Low harm	High harm	
Guilt in both the conditions (S1)	3(1–5)	6(5–7)	0.0002
Guilt (ALONE vs. TOGETHER)* (S2)	5(4–6)	6(5–7)	0.0006

Values indicate median (inter-quartile range [IQR]) of subjective ratings of guilt. *A high value indicates greater guilt in the ALONE condition.

In the ALONE condition, there were no significant correlations of TB effect with S1 (*ρ* = 0.29, *p* = 0.22) and S2 (*ρ* = 0.44, *p* = 0.06). In the TOGETHER condition, there was a significant correlation of TB effect with S1 (*ρ* = 0.49, *p* = 0.03), and a close-to-significant correlation of TB effect with S2 (*ρ* = 0.46, *p* = 0.05). A *post hoc* power analysis using G^∗^Power 3.1.9.2 was performed based on the current sample size and effect size, which indicated that the sample size was not enough to achieve a power of 80% at 5% alpha level.

Pre-session questionnaire responses allowed us to investigate whether causality attributions could be related to personality or trait empathy. Although we explored whether personality and empathy measures were related to the High-harm condition, the coefficients were generally weaker (Supplementary Data). A *post hoc* power analysis using G^∗^Power 3.1.9.2 was performed based on the current sample size and effect size, indicating that the sample size was not enough to achieve a power of 80% at 5% alpha level.

## Discussion

The purpose of this study was to investigate whether the presence of a co-actor changes causal attribution in situations where the causes of the outcomes were obvious. To examine this issue, causal attribution was measured by the TB method combined with a simple gambling task, and guilt was induced by giving a financially negative outcome to a third party. In the results of TB and the post-session emotion self-reports, there were significant differences between the Low-harm and the High-harm conditions; therefore, we believe that the procedure of the amount of money was appropriate for inducing guilt. In the High-harm condition, the TB effect was enhanced in the ALONE condition compared to in the Baseline condition; on the other hand, the TB effect was reduced in the TOGETHER condition. We suggest that the enhancement of perceived external attribution is influenced by the presence of a co-actor, even when one knows that the causes of the outcomes are obvious.

In a previous study of guilt (Yu et al., 2014), the responsibility attribution was observed in situations where it was obvious that both the participant and the co-actor were the causes of an outcome. The participants in that study completed multiple rounds of a dot-estimation task with anonymous partners. In the dot-estimation task, the partner would receive pain stimulation if the partner, the participant, or both responded incorrectly. The participant was then given the option to intervene and bear a proportion of pain for the partner. The participant felt more responsible when they participant was solely responsible for the stimulation (Self_Incorrect) than when they both committed an error (Both_Incorrect). However, the study was different from the present study in the following aspect: in Self_Incorrect, the partner participated in the action, but the error was obviously caused by the participant alone. Furthermore, it was different from the present study in terms of decision-making, compensation, and harming oneself. Based on this study, we suggest that only co-actor participation in the action may modulate responsibility attribution.

Our results in the ALONE condition supported a previous study in terms of a greater responsibility attribution for negative outcomes against another person (Li et al., 2018). On the other hand, another study demonstrated that TB was reduced when negative outcomes occurred against the self, consistent with self-serving bias (Takahata et al., 2012). It should be noted that the causal attribution changed depending on whether the object of the outcomes was the self or another. The reason that people do not to attribute responsibility to the self when negative outcomes occur is to prevent depression and maintain self-efficacy. However, there may be socially devastating consequences if people do not attribute negative outcomes to themselves. In order to develop a society, it is important to learn the causal relationship between actions and negative outcomes and utilize them in future actions.

However, interestingly, in the TOGETHER condition, the negative outcome was not attributed to the self. Beyer et al. (2017) argued that the presence of other agents may lead to diffusion of responsibility in a procedure in which the outcomes of the participants and others were independent. In our study, the only difference between the ALONE and TOGETHER conditions was whether the experimenter participated in pressing the key. That is, sharing the action with the co-actor was important to enhance external attribution. A plausible explanation for our results is that the presence of a co-actor may lead to diffusion of responsibility. A previous study reported that TB effects were reduced by authority coercion (Caspar et al., 2016). Another previous study reported that following a leader reduced TB effects (Pfister et al., 2014). Although others played the role of triggers in these previous studies, the current study showed that the TB effect was also reduced when the other was a co-actor. In particular situations, social organizations with leaders and co-actors may have become structures that can lead to diffusion and passing of responsibility. Because of the reduction of internal attribution, groups may tend to behave more aggressively (Meier and Hinsz, 2004) and immorally (Deschenes and Esbensen, 1999). Our study suggests that clarification of the cause may not be sufficient to deal with diffusion of responsibility. To avoid taking wrong actions by diffusion of responsibility, it may be necessary to take responsibility alone. However, as diffusion of responsibility has the effect of reducing stress (El Zein et al., 2019), it may be necessary to properly select the actions of alone or groups to manage the organization well. It might be necessary to induce proper causal attribution of actions and outcomes in the perceptual process to avoid inhumane actions.

Several possible objections should be considered. The first is the experimental procedure to induce guilt. Even when an action has an unpredictable negative outcome, society still attributes the action to the agent. Our result suggests that this social attribution is consistent with the perceptual experience of their causal attribution. However, our study did not have a decision-making process, which is an important factor in guilt (Wagner et al., 2012). The TB effect may have had different results if the participants themselves selected predictable outcomes rather than them being unpredictable. The relationship between guilt involving the decision-making process and causal attribution should be investigated in future studies.

Second, in the correlation analysis results, part of the conditions was significant. Based on the results of the power analysis, the sample size was insufficient. Therefore, we have to use caution when discussing any lack of correlation. Aside from sample size, there are two possible reasons for why there were no significant correlations under the other conditions. The first possibility is the bias due to the difference in those measurement points. Subjective ratings of guilt were performed after all trials had been completed; meanwhile, TB effect was measured in every trial. The second possibility may include factors other than guilt. One of other influential factors of binding effects may be motivation. Di Costa et al. (2018) reported that a binding effect is modulated in a context involving motivation using negative outcomes (error feedback). In the present study too, the decrease in motivation due to the presence of a co-actor might have reduced the causal attribution (binding effects).

Third, time-series analysis indicated that the participant’s guilt about the third party did not increase as the number of trials increases in the High-harm condition. Previous research has not investigated effects of the total amount of harm (Wagner et al., 2012). The time-series experimental design that accompanies the addition of harm will contribute to the development of this research field. Fourth, most of the results of the empathy questionnaire were not related to the TB effects. Because guilt and empathy are closely related (Baumeister et al., 1994), manipulation of empathy may modulate TB effects. However, based on the results of the power analysis, the sample size was insufficient; therefore, we must use caution when discussing the no relationship. Fifth, the generalizability of the conclusion may be limited because the majority of the participants were female. This limitation may affect the results on empathy. Finally, the influence of the amount of money should be considered. For the ethical consideration of participants, it was necessary to set a minimum amount of money to induce guilt. A preliminary experiment found that 200 yen was the most appropriate for causing guilt. Increasing amounts of money may affect guilt and responsibility.

## Conclusion

We investigated whether the presence of a co-actor changes causal attribution by using a TB method combined with a simple gambling task in situations where the causes of the outcomes were obvious. Our results showed that the perceptual internal attribution of actions and the outcomes was enhanced when people gave negative outcomes to a third party; on the other hand, external attribution was enhanced by the presence of the co-actor. Our study suggests that diffusion and passing of responsibility in social contexts involves modulation of perceptual causal attribution by participation of a co-actor. It might be necessary to induce proper causal attribution of actions and the outcomes in the perceptual process to avoid inhumane actions in social situations with co-actors.

## Data Availability Statement

The original contributions presented in the study are included in the article/Supplementary Materials, further inquiries can be directed to the corresponding author/s.

## Ethics Statement

The studies involving human participants were reviewed and approved by the Kio University ethics committee (R1-29). The patients/participants provided their written informed consent to participate in this study.

## Author Contributions

All authors contributed to the development and design of the studies. YN developed the experimental task. KH conducted the experiments and analyzed the data. KH drafted the manuscript. YM and SM provided critical revisions. All authors contributed to the article and approved the submitted version.

## Conflict of Interest

The authors declare that the research was conducted in the absence of any commercial or financial relationships that could be construed as a potential conflict of interest.
